# A Coupling Mechanism between Flicker Noise and Hot Carrier Degradations in FinFETs

**DOI:** 10.3390/nano13091507

**Published:** 2023-04-28

**Authors:** Minghao Liu, Zixuan Sun, Haoran Lu, Cong Shen, Lining Zhang, Runsheng Wang, Ru Huang

**Affiliations:** 1 School of Integrated Circuits, Peking University, Beijing 100871, China; 1801111284@pku.edu.cn (M.L.); szixuan_pku@126.com (Z.S.); 1900012734@pku.edu.cn (H.L.);; 2 School of Electronic and Computer Engineering, Peking University, Shenzhen 518055, China; 2201212772@stu.pku.edu.cn

**Keywords:** reliability, flicker noise, hot carrier degradation (HCD), compact model

## Abstract

A coupling mechanism between flicker noise and hot carrier degradation (HCD) is revealed in this work. Predicting the flicker noise properties of fresh and aged devices is becoming essential for circuit designs, requiring an understanding of the fundamental noise behaviors. While certain models for fresh devices have been proposed, those for aged devices have not been reported yet because of the lack of a clear mechanism. The flicker noise of aged FinFETs is characterized based on the measure-stress-measure (MSM) method and analyzed from the device physics. It is found that both the mean and deviations of the noise power spectral density increase compared with the fresh counterparts. A coupling mechanism is proposed to explain the trap time constants, leading to the trap characterizations in their energy profiles. The amplitude and number of contributing traps are also changing and are dependent on the mode of HCD and determined by the position of the induced traps. A microscopic picture is developed from the perspective of trap coupling, reproducing well the measured noise of advanced nanoscale FinFETs. The finding is important for accurate flicker noise calculations and aging-aware circuit designs.

## 1. Introduction

Flicker noise plays an important role in analog/RF circuit designs, such as in causing phase noise and jitter [1]. On the other hand, hot carrier degradation (HCD) is more severe than bias temperature instability (BTI) in analog/RF circuits, which is also one of the key reliability challenges for circuit design [2,3]. Previously, extensive experimental and theoretical studies have been conducted on flicker noise and HCD. Models which describe them independently have also been reported [4,5,6,7,8,9,10,11]. 

Considering that the HCD in advanced FinFETs is attributed to the interface states and traps in the dielectric [12,13,14], the flicker noise due to the inelastic trapping/de-trapping may interplay with the HCD-generated traps. In other words, the transistors of analog/RF circuits suffering from HCD may be subject to a change in the flicker noise property [15]. If the same flicker noise model of fresh devices is used under that condition, inaccuracy is foreseen in the circuit simulations, which leads to over-optimistic or over-pessimistic estimations of the circuit lifetime. However, in literature, studies on the possible impacts of HCD on flicker noise remain limited, although many reports on the individual phenomenon have been performed. This is partially attributed to flicker noise modeling from the traditional perspective, which does not involve the possible connections to HCD. As a result, it is urgent to examine the possible coupling between flicker noise and HCD and, if confirmed, to further reveal the mechanism.

In this paper, in order to solve the problem of the inaccurate prediction of flicker noise after HCD, the coupling effect between traps induced by HCD and traps which generate flicker noise is studied. The impacts of HCD traps on flicker noise in different Vgs/Vds regions are covered. HCD has an impact on the amplitude distribution and coupling strength of traps. The conventional model based on carrier number fluctuation theory and mobility fluctuation theory describes the worst-case using corner models. On the contrary, the statistical model considering the coupling effect between flicker noise and HCD agrees excellently with the measurement data of advanced technology nodes before and after HCD. With an improved accuracy compared with conventional models, the revealed coupling mechanism is meaningful in predicting flicker noise accurately in FinFET and Gate-All-Around (GAA) circuit designs [16].

## 2. Characterizing Flicker Noise of FinFETs

**Figure 1:**
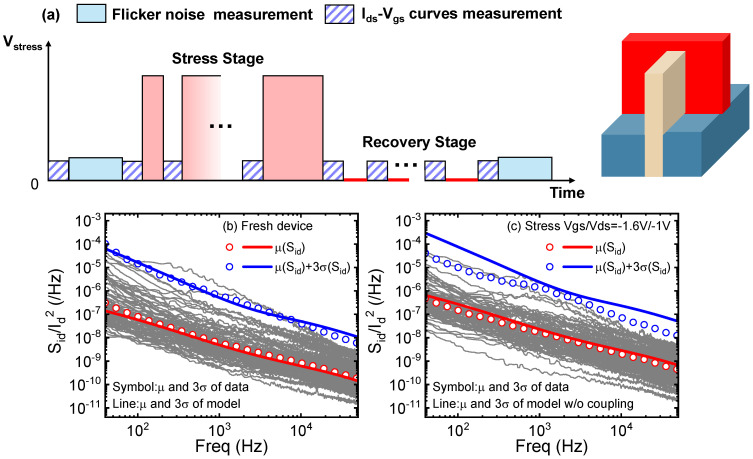
(**a**) The measure-stress-measure (MSM) method incorporating low-frequency noise characterizations and a schematic diagram of FinFET nanoscale structure; (**b**) statistical flicker noise properties of a fresh FinFET, and (**c**) the noise of the same FinFET after HCD. μ stands for the mean value and σ stands for the standard deviation.

Foundry-level 16/14 nm pFinFETs are used to explore the impacts of HCD on flicker noise. In this work, the classical measure-stress-measure (MSM) method is adopted by incorporating low-frequency noise characterizations for the experiment data. Figure 1a shows the measurement flow and a schematic diagram of the nanoscale FinFET structure. The main difference between the FinFET and planar MOSFET structure is that its channel is composed of tall and thin fins protruding from the insulating layer, which increases the surface of the gate around the channel and strengthens the control of the gate to channel. All the characterizations are performed with Agilent B1500A and Primarius-tech ProPlus 9812D in the authors’ research lab. To avoid the possible impacts of bias-temperature instability (BTI), which is mixed with the HCD [11], a recovery stage is implemented. The flicker noise characterizations are performed for fresh devices as well as aged devices (which refer to devices undergoing HCD for a certain period), with a fixed bias of Vgs/Vds = 0.4 V/0.05 V. Ids-Vgs curves are measured with Vds = 50 mV for the threshold voltage shifts of HCD.

Considering the two HCD modes, the single vibrational excitation (SVE) and multiple vibrational excitation (MVE) [17], and possibly different noise properties afterward, the stress voltages in the MSM are also varied in the characterizations. For the SVE mode, the stress voltage is designed with bias voltages Vgs/Vds = −1.0 V/−1.8 V. For the MVE mode, the stress voltage is chosen with Vgs/Vds = −1.6 V/−1.0 V. The recovery condition is Vgs = Vds = 0 V. The two HCD modes have been proven with different contributions of oxide traps, which do not recover [11].

The flicker noise properties of fresh and aged FinFETs with HCD of MVE are shown in Figure 1b,c as an example of the FinFET technology. Multiple devices are measured for the statistical noise, i.e., the mean and standard deviations. To exclude the influence of the current changes induced by HCD, the normalized noise power spectrum density (PSD) S_id_/I_d_^2^ at the given bias is plotted in grey lines, with the mean and deviations represented by the circles. It is seen that the aged devices suffer from severer flicker noise after HCD.

## 3. Analyzing the Flicker Noise

Considering the nature of flicker noise as the superposition of individual trap-induced noises [18], the frequency domain noise data could be analyzed interchangeably with the time domain data. In the time domain, the observable properties are the trap time constants such as the capture and emission time. Both the trap amplitude and time constants are extracted from time domain data with the method of the hidden Markov model (HMM) [19].

**Figure 2:**
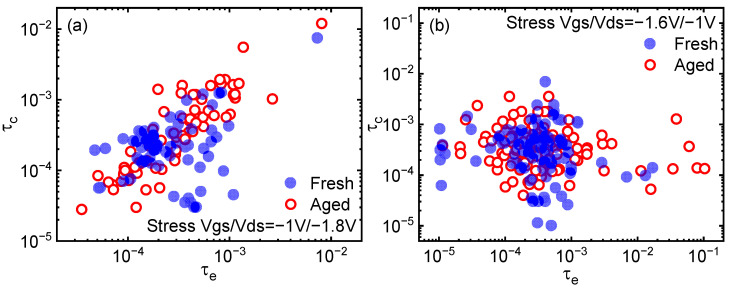
(**a**) The CET map before and after degradation under SVE stress bias condition. (**b**) The CET map before and after degradation under MVE stress bias condition. The time constant distribution remains almost unchanged after both stress bias condition.

The trap capture/emission time (CET) maps [20] are plotted in Figure 2, with both the SVE and MVE modes of HCD. The time constants of traps are not correlated to their amplitudes, allowing the study of major traps to cause “obvious” noise, i.e., random telegraph noise (RTN). At first sight, the CET map has no significant changes after both SVE and MVE stress bias conditions, which indicates that the time constant has a wide distribution to tolerate the threshold voltage shift induced by degradation. Differences in the CET maps of fresh devices are observed since FinFETs of different fin numbers are characterized for a complete picture. According to previous work and other BTI experimental studies [21], there can be two types of traps with different energy distributions that contribute to flicker noise. In this work, there is only one cluster of the distribution shown in the CET map. Because of the limitation of the measurement window, it is difficult to collect enough random telegraph noise (RTN) traps with small time constants to show another cluster of distribution.

**Figure 3:**
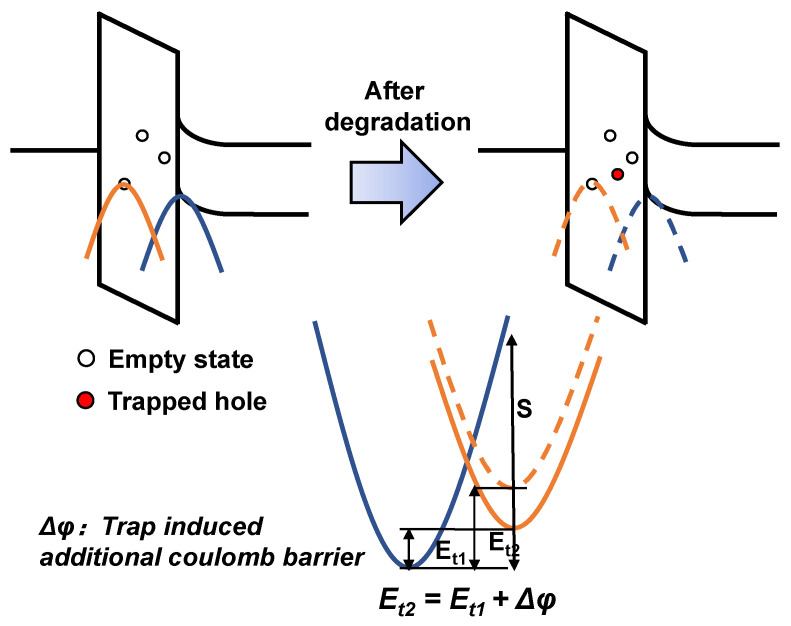
A proposal of coupling mechanism between the HCD-generated traps and those contributing to flicker noise. The positively charged trap may change the energy levels of noise traps through Coulombic interactions, leading to the change in trapping-de-trapping barriers.

At the same time, the fine modeling of the flicker noise reveals that the trap properties have been changed after the HCD. A coupling theory based on the inelastic trapping/de-trapping mechanism [22] is shown in Figure 3. The model is semi-classical, and the energy of traps and carriers is considered to be in the form of a parabola. Currently, the energy barrier ΔEB is not only determined by the lowest energy of flicker noise traps and carriers; it also needs to consider the influence of relaxation energy S. For the fresh devices, carriers in the channel interact with the traps in the gate dielectric with the assistance of multi-phonons, which is different from the traditional elastic tunneling theory. While the HCD-induced traps with deep levels do not recover and do not contribute directly to the flicker noise, the charged traps potentially affect the energy levels of noise-related traps because of the Coulombic interactions.

At the microscopic level, three parameters are associated with each trap, including the trap amplitude ΔId, trap energy E_t_, and the relaxation energy S. In the inelastic tunneling theory, the trap time constant τ is determined by [18]:

(1) $$\tau =\frac{1}{{\mathrm{nv}}_{\mathrm{th}}\sigma_{0}}\exp\left(\frac{\Delta E_{B}}{k_{B}T}\right)$$

(2) $$\Delta E_{B}=\frac{{\left(S+E_{f}+\phi_{S}-E_{t}\right)}^{2}}{4S}$$

where n, v_th_, σ_0_, Δ*E_B_*, *E_f_*, and $\phi_{S}$ are the number of carriers, the thermal velocity of carriers, capture cross-section activation energy of traps, Fermi level, and surface potential, respectively. The number of carriers and the surface potential are considered based on the structure of FinFET. Two types of traps are observed in the fresh devices, with separate distributions of the energy levels and relaxation energies, both following the Gaussian distribution.

**Figure 4:**
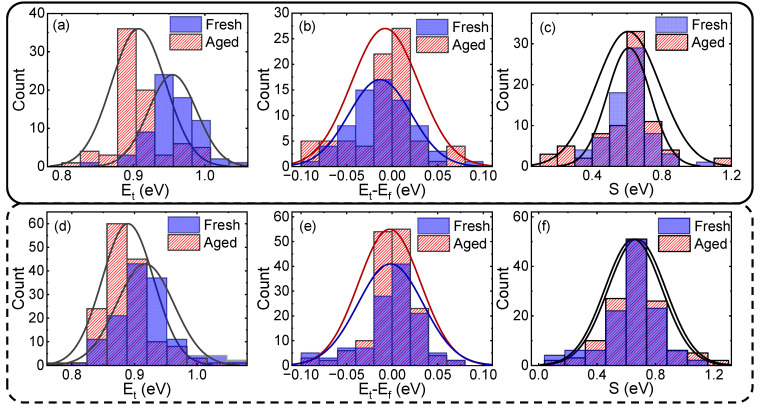
(**a**–**c**) are trap energy distribution, relative trap energy distribution, and relaxation energy distribution before and after SVE stress bias condition. (**d**–**f**) are trap energy distribution, relative trap energy distribution, and relaxation energy distribution before and after MVE stress bias condition.

Based on this, we can extract energy distributions from the CET map in Figure 2. The results are shown in Figure 4. The trap energy E_t_ changes after degradation, while the time constant distribution is almost unchanged. From Equation (2), the activation energy Δ*E_B_* is determined by the relative energy level *E_t_–E_f_* and relaxation energy S. The HCD not only changes the trap energy distribution but also induces a threshold voltage shift. As a result, the relative trap energy distribution remains almost unchanged, as shown in Figure 4b,e. The impact of HCD on relaxation energy distribution is shown in Figure 4c,f. It can be seen that the HCD has little impact on relaxation energy. This is consistent with the assumption that HCD mainly affects trap energy distribution.

**Figure 5:**
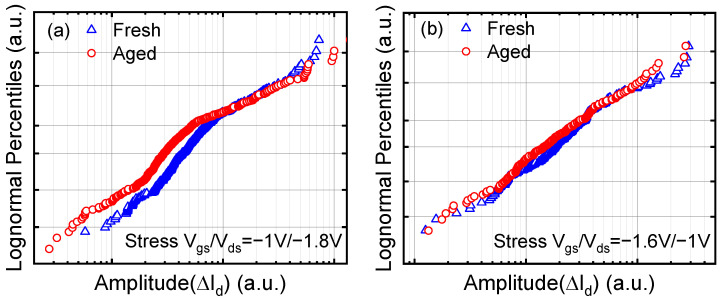
(**a**) The distribution of amplitude before and after SVE stress bias condition. (**b**) The distribution of amplitude before and after MVE stress bias condition.

The amplitude (ΔId) distribution before and after degradation is shown in Figure 5, following the lognormal distribution function. HCD of the SVE type has a more significant impact on the trap amplitude than that of the MVE type. The devices have less degradation after the MVE stress bias condition, and the distribution of amplitude remains almost unchanged. This is because the MVE stress bias condition is more similar to the negative bias temperature instability (NBTI) stress bias condition, and most of the degradation recovers under recovery bias.

Summing the total number of traps together with the individual contribution to the noise spectrum, we reproduce the flicker noise property, including both the mean and standard deviation. While each trap contributes to noise as a Lorentzian-type spectrum, the summation may present the 1/f characteristics when the number of traps is large or the mixed 1/f and 1/f^2^ characteristics. From the flicker noise modeling [18], the number of traps follows a Poisson distribution, and the trap statistics are essential.

**Figure 6:**
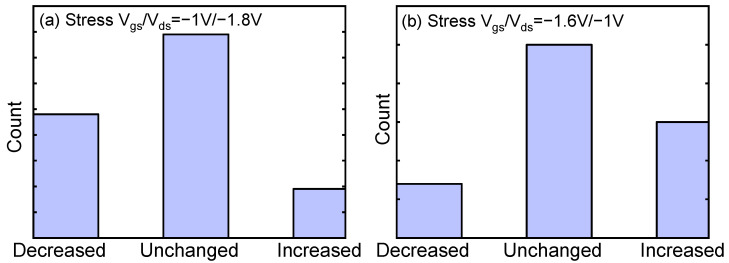
(**a**) The change in number of traps before and after SVE stress bias condition. (**b**) The change in number of traps before and after MVE stress bias condition.

The trap number in each device is extracted by combining the frequency and time domain characterizations. The same devices are characterized to check the possible changes in the trap number after HCD. The coupling effect between the traps induced by HCD and the traps that generate flicker noise will lead to a change in the coupling strength between the flicker noise traps. This will lead to changes in the amplitude distribution of flicker noise traps, which may change the number of RTN traps. Therefore, the coupling effect between the traps induced by HCD and the traps which generate flicker noise can be studied by the change in the number of RTN traps. Figure 6 summarizes the change in the number of RTN traps, which categorizes the aged FinFETs into three groups: the group with decreased number of traps, the group with an unchanged number, and the group with an increased number.

It shows the opposite trend under different bias conditions. In most devices, the number of traps remains unchanged after both the SVE and MVE stress bias conditions. After the SVE stress bias condition, more devices with a decreased number of traps are observed as compared with those with an increased number of traps. On the contrary, after the MVE stress bias condition, there are more devices with an increased number of traps contributing to noise as compared with those with a decreased number of traps.

**Figure 7:**
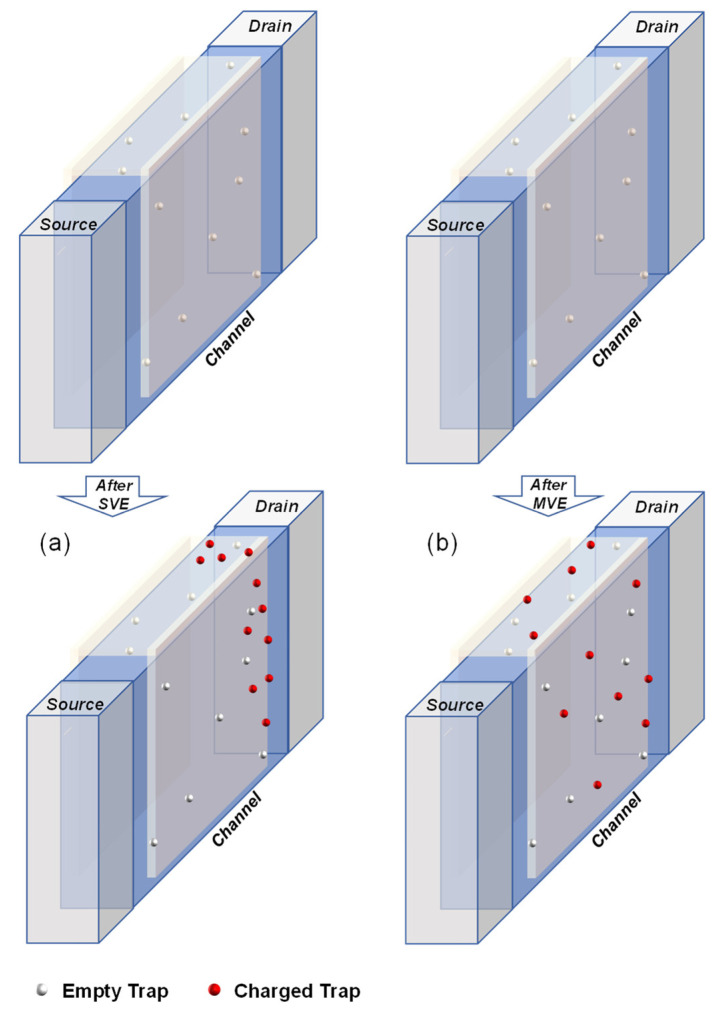
Schematic diagram of the location of new generated trap after (**a**) SVE stress bias condition and (**b**) MVE stress bias condition.

The change in the number of traps and the amplitudes are indeed correlated to the coupling effect between traps. Traps induced by different bias stress conditions have different impacts on the coupling. This is because of the different locations of the new traps generated by different processes [23]. The trap amplitude negatively correlates with the coupling strength. As shown in Figure 7, under the SVE bias condition, most of the hot carriers which gain enough energy from the electric field are near the drain region. This means that the generated traps are mainly concentrated at the drain side of the device, which increases the coupling between traps. On the contrary, after the MVE bias condition, as Vgs is large, the number of carriers plays a key role in generating traps. This means that the generated traps have a more uniform distribution in the channel, and this can decrease the coupling strength between traps.

## 4. Flicker Noise with the Coupling Mechanism

With the above evidence of trap coupling, the flicker noise properties of FinFETs after HCD should be understood by incorporating the coupling effects in the statistical model.

**Figure 8:**
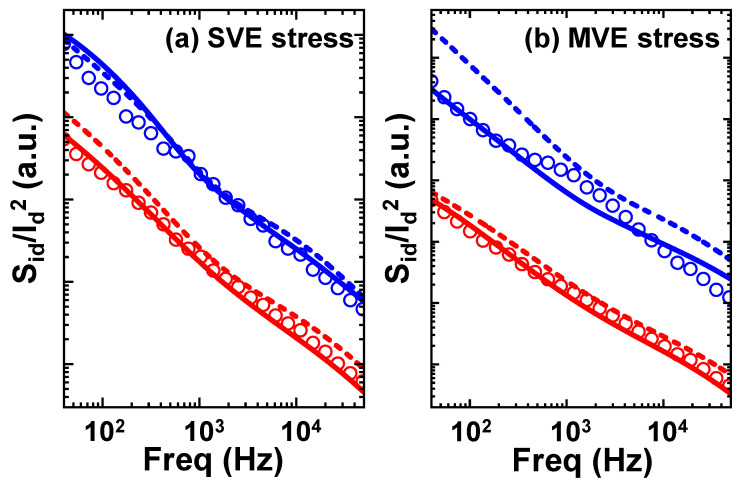
Comparisons of measurement results and model. (**a**) aged devices after SVE stress bias condition; (**b**) aged devices after MVE stress bias condition. Symbols stand for experimental data, and solid lines represent simulation results with coupling effect. Dashed lines stand for simulation results without coupling effect. Red and blue symbols are the mean and standard deviations (three sigma), respectively.

Figure 8 shows measurement results together with the previously proposed statistical model under the SVE bias condition and MVE bias condition, with and without coupling. It can be seen that the results that consider the coupling effect agree better with the data. The energy distribution parameters are based on those extracted from the measurement data in Figure 4. The amplitude distribution parameters are directly fitted with measurement data with the lognormal distribution. There is still a small deviation in the model, for example, in Figure 8b. We speculate that in addition to RTN traps with large amplitudes, there are traps with smaller amplitudes that contribute to the flicker noise. These traps need to be included further in the statistical model. Table 1 is given to show the differences between the conventional model and the statistical flicker noise model adopted in this work.

## 5. Conclusions

In this work, the flicker noise properties of FinFETs after HCD are studied. The characterizations and analysis show that the time constants of the associated traps are not changing significantly, in contrast to their amplitudes, especially with the SVE-type stress. These results suggest the importance of trap coupling between the HCD-induced traps and those contributing to the flicker noise. Changes in the number of traps are also understood with the coupling theory. With these findings, the flicker noise of aged FinFETs could be well reproduced. Based on this conclusion, a complete reliability model covering the coupling mechanism could be further developed, and the flicker noise properties in the next-generation GAA technology could also be further studied.

## Figures and Tables

**Table 1:** Comparison of conventional model and statistical model with and without coupling.

	Conventional Model	Statistical Model	Statistical Model with Coupling
Theoretical basis	Number fluctuation theory and mobility fluctuation theory	Inelastic trapping/de-trapping with the assistance of multi-phonons	Inelastic trapping/de-trapping with the assistance of multi-phonons
Key factors	Trap energy and trap position	Trap energy, relaxation energy, and amplitude	Trap energy, relaxation energy, and amplitude
Method of reflecting variations	Corner model	Monto-Carlo method	Monto-Carlo method
Whether to reflect RTN feature	No	Yes	Yes
Whether to consider the coupling effect between flicker noise and HCD	No	No	Yes

## Data Availability

Data sharing is not applicable to this article.
